# Effect of Player Role and Competition Level on Player Demands in Basketball

**DOI:** 10.3390/sports9030038

**Published:** 2021-03-08

**Authors:** Jodie Palmer, Daniel Wundersitz, Rodrigo Bini, Michael Kingsley

**Affiliations:** 1 Holsworth Research Initiative, La Trobe Rural Health School, La Trobe University, Bendigo 3552, Australia; jodie.palmer@latrobe.edu.au (J.P.); d.wundersitz@latrobe.edu.au (D.W.); r.bini@latrobe.edu.au (R.B.); 2 Department of Exercise Sciences, Faculty of Science, University of Auckland, Auckland 1023, New Zealand

**Keywords:** accelerometry, relative exercise intensity, athlete monitoring, time-motion analysis, training, matches

## Abstract

This study compared basketball training and match demands between player roles (starters, in-rotation bench players, out-rotation bench players) and between competition levels (semi-professional, professional). Thirty-seven players from one professional women’s team, one semi-professional women’s team, and one semi-professional men’s team wore accelerometers during training and matches throughout a competitive season. All teams were used for player role comparisons and the women’s teams were used to compare competition levels. Match and training session average intensity and volume, and durations of relative exercise intensities (inactive, light, moderate-vigorous, maximal, supramaximal) were calculated. Compared to out-rotation bench players, starters experienced twice the average match intensity and volume, spent 50% less match time being inactive, and spent 1.7–4.2× more match time in all other activity categories (*p* < 0.01). Compared to in-rotation bench players, starters experienced 1.2× greater average match intensity and volume, spent 17% less match time being inactive, and spent 1.4–1.5× more match time performing moderate-vigorous and maximal activity (*p* < 0.01). No differences in match demands were found between women’s competition levels, however the professional team experienced double the cumulative weekly training volume of the semi-professional team and spent 1.6–2.1× more cumulative weekly time in all activity categories (*p* < 0.01). To improve performance and reduce injury risk, players should prepare for the greatest match demands they could encounter during a season while considering potential changes to their role. Additionally, players might need their training volume managed when transitioning from a semi-professional to a professional season to reduce the injury risk from sharp increases in training demands.

## 1. Introduction

Possessing an adequate level of physical conditioning is crucial for optimum performance and injury prevention in basketball [1,2]. To achieve adequate physical conditioning, training demands should meet or exceed the demands of competition. Various methods have been used previously to quantify the movement demands of basketball (e.g., time-motion analysis), and the physiological (e.g., heart rate, blood lactate) and perceptual (e.g., rating of perceived exertion) responses to those demands. Recently, accelerometry has been proposed as a useful tool for quantifying the movement demands of basketball [3]. While extensive research to quantify match demands of basketball has been conducted and reviewed [4], match demands can vary between players on the same team. In professional women’s basketball, starters performed less sedentary and more vigorous-intensity activity than bench players during matches across a professional season [5], which is intuitive because starters generally receive more playing time [5,6,7,8].

While starters and bench players might seem a logical form of player categorization, it might not capture the range of roles that players perform in a basketball team. Teams are permitted to use 12 players in a match, but coaches often establish a 7–8 player rotation [9]. This means the top 7–8 players receive substantial playing time while the remaining players receive minimal, where some players average <1 min per game across a season [5]. It has previously been suggested that players who train once or twice a week and play minimal minutes in matches might not receive sufficient stimuli to maintain adequate conditioning in-season [10]. Comparing the demands of all bench players with starting players might overestimate the match demands of bench players who do not play in the main rotation, and therefore underestimate the additional conditioning stimulus required to maintain their conditioning levels [10]. This issue would be amplified if bench players not in the main rotation also experience lesser demands in team training than other players. Subsequently, comparisons of match demands of bench players not in the main rotation with starters and bench players in the main rotation is warranted and has not yet been performed.

In addition to match demands differing between starters and bench players, match demands have also differed between levels of competition [11,12]. In men’s basketball, studies have agreed that higher levels of competition elicit more moderate-intensity activity but contradict on which level elicits more high-intensity activity [11,12]. It is suggested that tactical factors and differences in individual skill levels contribute to the differences in match demands between competition levels [11,12].

Knowledge of differences in match demands between competition levels is important to adequately prepare players transitioning from lower to higher levels of competition. These transitions occur in development pathways when national-level players are selected onto international teams, when amateur players play their first semi-professional season, and when semi-professional players play their first professional season. Additionally, in women’s basketball, players often play semi-professionally in the off-season of their professional competition to supplement their income, which is typically lower than in men’s basketball [13]. In this instance, knowing differences in training and match demands can guide the management of training and match demands at the inter-season transition points to ensure players do not experience a large spike in activity demands and a subsequent increase in injury risk [2]. While differences in match demands between competition levels have been shown to exist in men’s basketball [11,12], no study to date has investigated this in women’s basketball. This investigation is required specifically in women’s basketball due to match demands differing between men’s and women’s basketball, preventing findings from men’s basketball being transferred to women’s basketball [14]. Additionally, no study to date has investigated differences in training demands between competition levels, where this information could complement the knowledge of differences in match demands and further assist players to prepare for training and competing at a higher level. Therefore, more investigation is required to conclude how match and training demands in basketball differ between competition levels.

Basketball training and match demands have typically been quantified using video-based time-motion analysis [11,12], where absolute velocity thresholds are used to quantify on-court activity. As basketball is a highly intermittent sport involving frequent changes of direction and accelerations, quantifying activity by velocity thresholds might not be appropriate, particularly when categorizing high accelerations from low starting speeds [15]. Additionally, the use of absolute thresholds does not consider the differences in effort required to move at the same pace for different players. While the recent use of accelerometry-derived PlayerLoad™ to quantify activity, demands overcomes some of these issues, a limitation is the large between-subjects variations in magnitude, making it an unsuitable metric for comparing demands between players [16]. An accelerometry-derived force metric and associated method of calculating relative intensities has recently been developed and validated [5,17]. Due to its advantages over other methods and its recent use in basketball, this method is used to quantify training and match demands in the present study.

The aims of this study were twofold: 1) investigate differences in training and match demands between player roles in basketball, and 2) investigate differences in training and match demands between semi-professional and professional women’s basketball.

## 2. Materials and Methods

### 2.1. Participants

Basketball players from one semi-professional men’s team, one semi-professional women’s team, and one professional women’s team (Table 1) belonging to the same basketball organization participated in the study. The semi-professional teams competed in the Australian 2019 NBL1 season (the second-highest level of men’s and women’s competition in Australia), and the professional team competed in the Australian 2019/20 WNBL season (the highest level of women’s competition in Australia). Players provided written informed consent prior to participating. Ethical approval was granted by the La Trobe University Human Research Ethics Committee (HEC15-088) in accordance with the Declaration of Helsinki.

### 2.2. Procedures

Movement activity was monitored during in-season on-court team training sessions and matches (Table 1). The semi-professional teams trained 1,2 times a week for 1.5–2 h per training, while the professional team trained 2,3 times a week for 1.5–2.5 h per training. During the team training sessions and matches, players wore a 100 Hz triaxial accelerometer (GT9X Actigraph, Pensacola, FL, USA) in a pouch on a tightly fitted sports vest, with the accelerometer positioned between the athlete’s scapulae [18]. Accelerometers have shown acceptable intra- and inter-device reliability in laboratory and team sport settings [19,20,21,22].

Accelerometer data from on-court team training sessions were recorded from the beginning to the end of the session. For matches, accelerometer data were obtained for all activity from the beginning of the first quarter to the end of the final quarter, inclusive of stoppages, time-outs and inter-quarter breaks for all players who took the court. Manufacturer software (Actilife v6.13.4; Actigraph, Pensacola, FL, USA) was used to download raw accelerometer data, and data were processed using custom code in MATLAB (R2018b; MathWorks, Natick, MA, USA).

Prior to the respective season commencements, players participated in a testing session where body mass and stature were measured [23]. In the same testing session, players who were injury-free during the pre-season period (12 semi-professional men, eight semi-professional women, 11 professional women) also completed a modified Yo-Yo Intermittent Recovery 1 (IR1) Test while wearing the accelerometer to estimate the net resultant force exerted while walking and running at varying speeds and subsequently calculate relative exercise intensity [5]. Therefore, while minutes played, AvF_NET_ and Impulse data were available for all participants (n = 37), relative exercise intensity data were only available for participants who completed the Yo-Yo IR1 test (n = 31).

### 2.3. Data Analysis

Raw accelerometer data were filtered using a fourth-order band-pass Butterworth filter in MATLAB (MathWorks, Natick, MA, USA) with cut-off frequencies of 0.1 Hz and 15 Hz [17]. To quantify exercise intensity as average net resultant force (AvF_NET_; Equation (1)), accelerometer-derived average resultant acceleration for each session was multiplied by the player’s body mass. Session volume was quantified by multiplying the average session intensity by the session duration in seconds. These metrics have demonstrated construct validity in basketball when compared to predicted AvF_NET_ calculated from 2D movement speeds in a basketball exercise simulation test [17]. Additionally, these metrics have demonstrated stronger relationships with running speed than PlayerLoad™ per minute [17].

(1) $${\mathrm{AvF}}_{\mathrm{NET}}=\frac{\sum_{0}^{n}\left(\mathrm{BM}\right)\left(\sqrt{A_{x}{}^{2}+A_{y}{}^{2}+A_{z}{}^{2}}\right)}{n}$$

Equation (1): Average net force output, BM = body mass, A_x_ = acceleration in the x direction, A_y_ = acceleration in the y direction, A_z_ = acceleration in the z direction, n = number of 1-s epoch samples

Individualized relative exercise intensity bands were calculated using methods validated previously [5]. Specifically, linear relationships were developed between the individual players’ average net resultant force (AvF_NET_) during varying walking and running speeds and the estimated oxygen consumption (${\dot{V}O}_{2}$) at those same speeds [5]. To calculate these individual relationships between AvF_NET_ and ${\dot{V}O}_{2}$, players’ average net force outputs during each speed of the Yo-Yo IR1 were initially calculated [5]. Estimated ${\dot{V}O}_{2}$ during each speed of the Yo-Yo IR1 was then calculated using established relationships between linear movement speed and ${\dot{V}O}_{2}$ published in the ACSM guidelines [5,24]. AvF_NET_ was plotted against estimated ${\dot{V}O}_{2}$ for each speed of the Yo-Yo IR1 to determine individualized linear relationships for each player between AvF_NET_ and estimated ${\dot{V}O}_{2}$. Although the relationships are developed by performing predominantly forwards linear movement, previous research has shown that AvF_NET_ is able to quantify the demands of different lateral, backwards, vertical, and nonlinear movements such as shuffling, jumping, and changing direction. AvF_NET_ was higher for these movement demands than for forwards linear movement, enabling the appropriate categorization of all types of activity [17]. This method allows for movement demands to be quantified relative to the individual player’s movement characteristics and fitness levels, and subsequently enables comparisons of demands between individual players. Additionally, the use of established relationships between linear movement speeds and ${\dot{V}O}_{2}$ allows individual relationships between AvF_NET_ and ${\dot{V}O}_{2}$ to be determined in a team setting without the need for direct measurement of ${\;\dot{V}O}_{2}$. It is important to note that this method does not estimate oxygen consumption, but rather estimates exercise intensity using equivalent estimated oxygen consumption as a reference for categorizing activity intensity.

These individual linear relationships were then used to calculate relative exercise intensity bands based on percentage ${\dot{V}O}_{2}$ reserve (${\dot{V}O}_{2}$R). Intensity bands were defined as inactive, light, moderate-vigorous, maximal, and supramaximal. ${\dot{V}O}_{2}$R thresholds for each intensity band and average associated AvF_NET_ thresholds are displayed in Table 2. The upper threshold for light, and both thresholds for moderate-vigorous and maximal activity were defined according to ACSM recommendations [25]. The supramaximal category was added to encompass activity performed above 100% ${\dot{V}O}_{2}$R. An inactive band was added to encompass activity where players were not actively moving about the court. The upper threshold for the inactive band was set as 10% ${\dot{V}O}_{2}$R due to it representing very slow linear movement speeds (≤3.4 km·h^−1^) for the participants of this study. Average session intensity and volume, and absolute (min) and relative (% of session time) time spent in each intensity band were reported for team training sessions and matches throughout the season.

For the Part A analyses, comparisons of training and match demands were made between player roles. Roles were defined as starters (players who started the match on the court), in-rotation bench players (bench players who played 10 or more minutes in a match), and out-rotation bench players (remaining bench players), where minutes played data were retrieved from league websites [9,26,27]. For each role that a player performed throughout the season, their season-average match demands while performing that role were calculated. For comparing training session demands, players were classified as the role they most often played throughout the season. For the Part B analyses, comparisons of training and match demands were made between semi-professional and professional female players. Total weekly volume of activity performed by the semi-professional and professional team was also determined. Total weekly impulse was calculated by summing the impulse for each training session in a week. Total weekly duration spent in each intensity band was calculated by summing the durations from each training session in a week.

### 2.4. Statistical Analyses

Shapiro-Wilk tests indicated that several variables violated the assumption of normality. Data were therefore expressed as median (lower bound-upper bound). Nonparametric tests were conducted to determine differences in training and match demands between player roles, and differences in training demands between competition levels. Kruskal–Wallis H tests were conducted to determine the effect of player role on training and match demands, with Mann–Whitney U tests conducted using Bonferroni-Holm-adjusted *p*-values to determine where differences occurred. Mann-Whitney U tests were conducted to determine differences in training demands between competition levels. To control for minutes played, comparisons of match demands between competition levels were conducted using an Analysis of Covariance (ANCOVA) with log-transformed variables. Significance was set at *p* ≤ 0.05. Statistical analyses were conducted using SPSS Statistics (v26; IBM Corporation, Armonk, NY, USA). Effect sizes (ES) were calculated on log-transformed data and are presented as Cohen’s d, categorized as follows: <0.2: trivial, 0.2–0.6: small, >0.6–1.2: moderate, >1.2–2.0: large, and >2.0: very large [28].

## 3. Results

### 3.1. Part A

**Figure 1:**
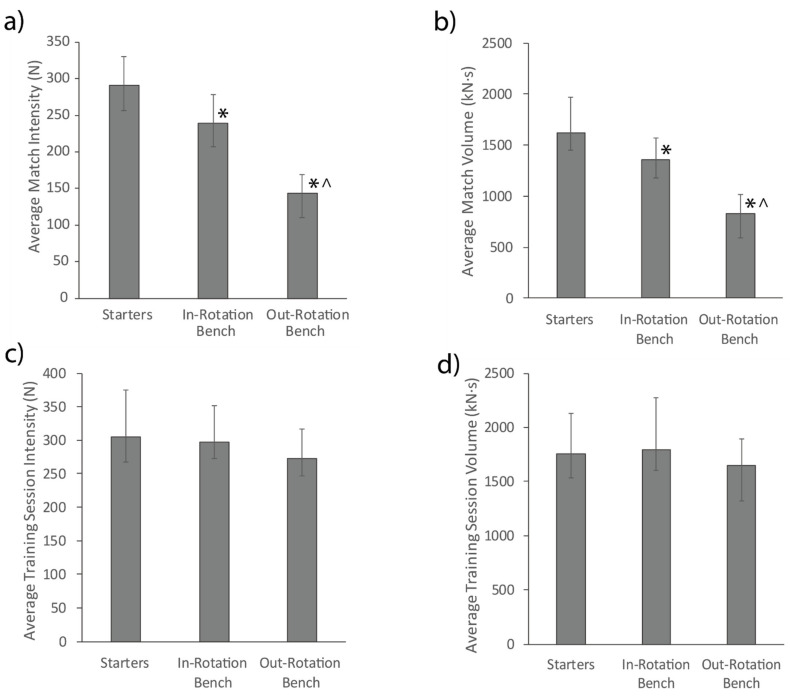
Average match intensity (**a**) and volume (**b**), and average training session intensity (**c**) and volume (**d**) for each player role. * represents a significant difference to starters (*p* < 0.05). ^ represents a significant difference to in-rotation bench players (*p* < 0.05). Error bars represent the upper and lower quartiles.

During matches, starters received more playing time (28.2 min [23.1–29.9 min]) than in-rotation bench (17.9 min [14.5–22.3 min]; ES: 1.28) and out-rotation bench (5.0 min [4.2–5.8 min]; ES: 1.98) players (*p* < 0.01), while in-rotation bench players also received more playing time than out-rotation bench players (*p* < 0.01, ES: 1.87). Average match and training intensity and volume for each player role are presented in Figure 1. In matches, starters had a greater average intensity and volume than in-rotation bench (intensity: ES: 0.96; volume: ES: 0.84) and out-rotation bench (intensity: ES: 1.76; volume: ES: 1.70) players, while in-rotation bench players also had a greater average intensity (ES: 1.87) and volume (ES: 1.55) than out-rotation bench players (*p* < 0.01). In training sessions, no differences occurred between player roles for average session intensity and volume (*p* > 0.05).

**Figure 2:**
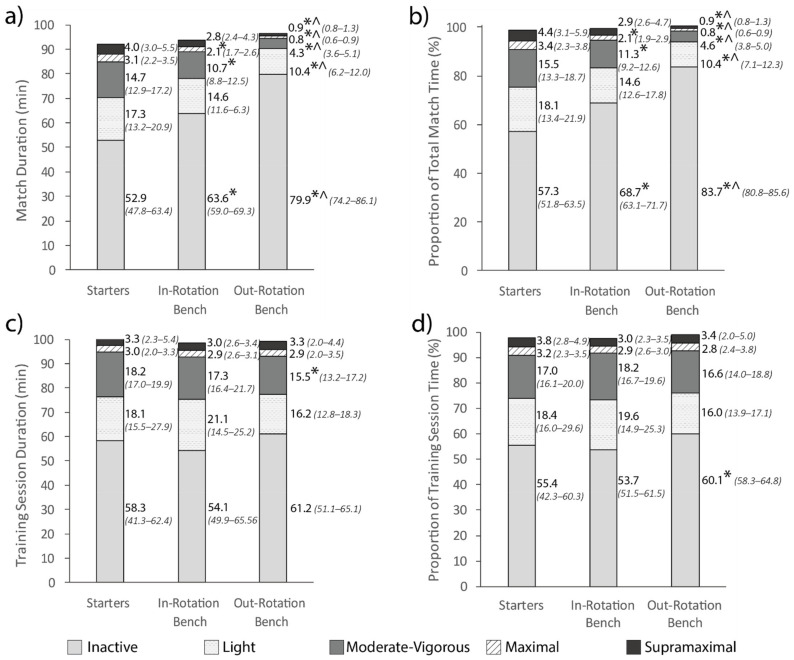
Total duration (**a**) and proportion of session time (**b**) spent in each intensity band in matches for each player role, and total duration (**c**) and proportion of session time (**d**) spent in each intensity band in training sessions for each player role. Adjacent values are presented as median (lower bound-upper bound). * represents a significant difference to starters (*p* < 0.05). ^ represents a significant difference to in-rotation bench players (*p* < 0.05).

Durations in each intensity band during matches and training sessions for each role are presented in Figure 2. During matches, out-rotation bench players spent more time being inactive than starters (ES: 1.49) and in-rotation bench players (ES: 1.36), while starters (light: ES: 1.29; moderate-vigorous: ES: 1.89; maximal: ES: 1.80; supramaximal: ES: 1.70) and in-rotation bench players (light: ES: 0.96; moderate-vigorous: ES: 1.70; maximal: ES: 1.72; supramaximal: ES: 1.59) spent more time in all other activity categories than out-rotation bench players (*p* < 0.01). Additionally, starters spent less time being inactive (ES: 0.84) and more time performing moderate-vigorous (ES: 1.12) and maximal (ES: 0.82) activity than in-rotation bench players (*p* < 0.01). During training sessions, out-rotation bench players spent less time performing moderate-vigorous activity than starters (*p* < 0.01, ES: 1.13), while no differences between player roles were seen for durations in all other intensity bands.

Proportions of session time spent in each activity category during matches and training sessions for each role are presented in Figure 2. During matches, out-rotation bench players spent a greater proportion of session time being inactive than starters (ES: 1.64) and in-rotation bench players (ES: 1.51), while starters (light: ES: 1.29; moderate-vigorous: ES: 1.88; maximal: ES: 1.76; supramaximal: ES: 1.69) and in-rotation bench players (light: ES: 0.97; moderate-vigorous: ES: 1.72; maximal: ES: 1.70; supramaximal: ES: 1.59) spent a greater proportion of session time in all other activity categories (*p* < 0.01). Starters also spent a lesser proportion of match time being inactive (*p* < 0.01, ES: 1.07) and a greater proportion of match time performing moderate-vigorous (*p* < 0.01, ES: 1.08) and maximal (*p* = 0.01, ES: 0.74) activity than in-rotation bench players. During training sessions, out-rotation bench players spent a greater proportion of session time being inactive than starters (*p* = 0.01, ES: 0.96), while no differences between player roles were seen for proportion of session time spent in all other intensity bands.

### 3.2. Part B

**Figure 3:**
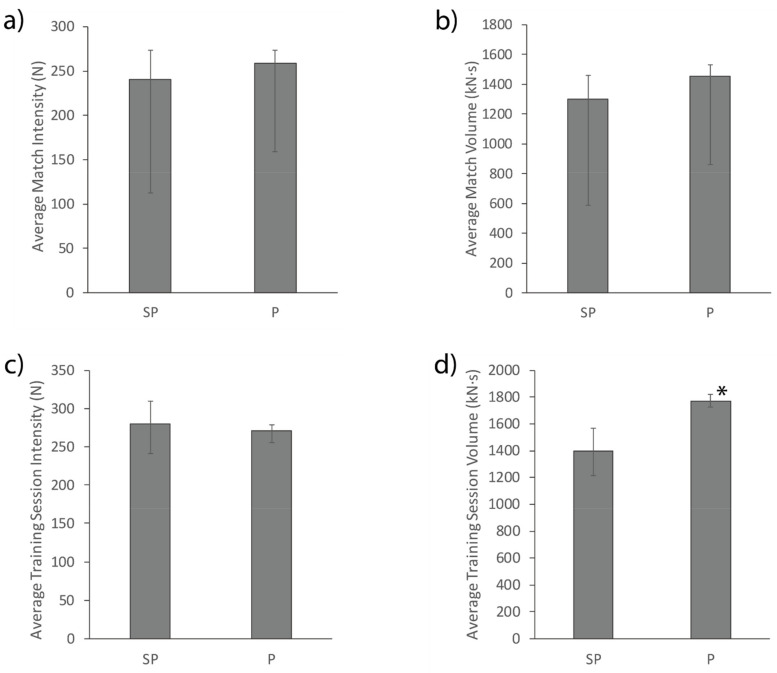
Average match intensity (**a**) and volume (**b**), and average training session intensity (**c**) and volume (**d**) for the semi-professional (SP) and professional (P) team. * Represents a significant difference to the semi-professional team (*p* < 0.05). Error bars represent the upper and lower quartiles.

Average match and training intensity and volume for the semi-professional and professional team are presented in Figure 3. In matches, no differences were found between the semi-professional and professional team for average intensity or volume (*p* > 0.05). In training sessions, the average volume was greater for the professional team than the semi-professional team (*p* < 0.01, ES: 1.17), while no differences were seen for average intensity (*p* > 0.05).

**Figure 4:**
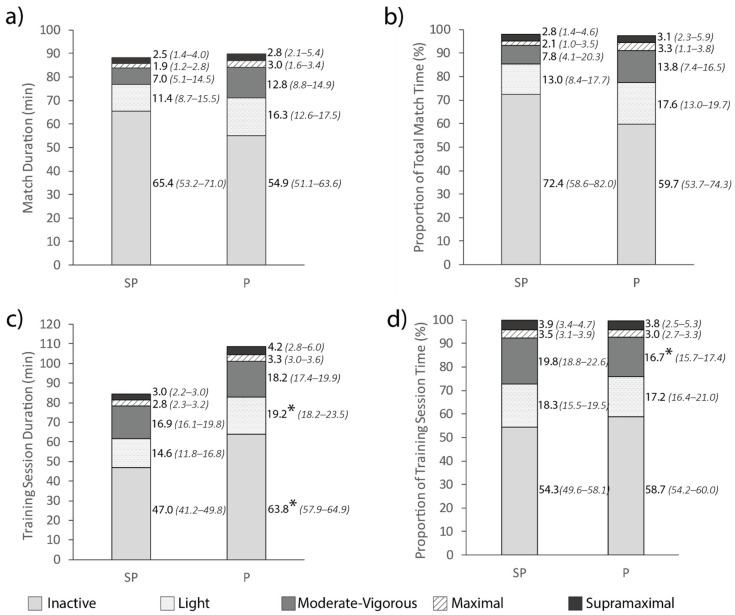
Total duration (**a**) and proportion of session time (**b**) spent in each intensity band in matches for the semi-professional (SP) and professional (P) team, and total duration (**c**) and proportion of session time (**d**) spent in each intensity band in training sessions for the semi-professional (SP) and professional (P) team. Adjacent values are presented as median (lower bound-upper bound). * Represents a significant difference to the semi-professional team (*p* < 0.05).

Durations in each intensity band during matches and training sessions for the semi-professional and professional team are presented in Figure 4. No differences were seen between competition levels for any match demands (*p* > 0.05). In training sessions, the professional team spent more time being inactive (*p* < 0.01, ES: 1.43) and performing light intensity activity (*p* = 0.01, ES: 0.97), while the semi-professional team spent a greater proportion of training session time performing moderate-vigorous activity (*p* < 0.01, ES: 1.21).

**Figure 5:**
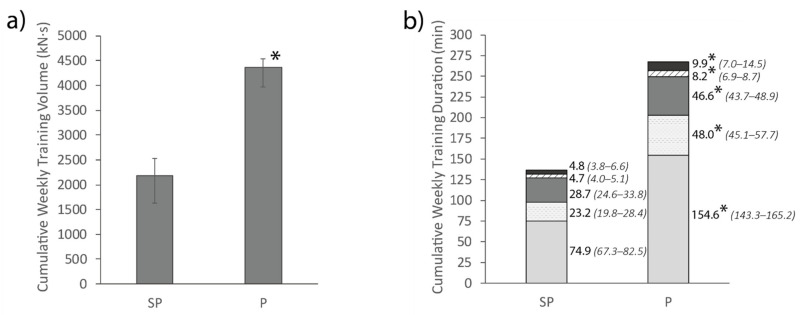
Average total weekly training volume (**a**) and total duration spent in each intensity band (**b**) for the semi-professional (SP) and professional (P) team. Adjacent values are presented as median (lower bound-upper bound). * Represents a significant difference to the semi-professional team (*p* < 0.05).

Cumulative weekly training volume and cumulative weekly duration in each intensity band for the semi-professional and professional team are presented in Figure 5. The professional team had a greater average cumulative weekly training volume than the semi-professional team (*p* < 0.01, ES: 1.72). The professional team also spent more cumulative weekly training time in all activity categories (inactive: ES: 1.76; light: ES: 1.59; moderate-vigorous: ES: 1.62; maximal: ES: 1.64; supramaximal: ES: 1.34) than the semi-professional team (*p* < 0.01).

## 4. Discussion

Findings from this study revealed that: (1) starters experienced greater match demands than both in-rotation and out-rotation bench players, and in-rotation bench players experienced greater match demands than out-rotation bench players, (2) minimal differences existed between player roles for training demands, and (3) when comparing competition levels, no differences in match demands were present, while differences in both sessional and cumulative weekly training demands were found.

Differences in match demands between starters, in-rotation bench players and out-rotation bench players were found for multiple variables (Figure 1 and Figure 2). Starters spent less absolute and relative time being inactive and performed more absolute and relative moderate-vigorous and maximal activity in matches than in-rotation bench players without differences in supramaximal activity, despite starters playing more minutes. This result suggests starters might pace themselves during matches, similar to other team sports [29]. These findings reflect previous research in women’s basketball, where starters performed less sedentary and more vigorous activity than bench players, with no differences for supramaximal activity, but show a difference in maximal activity not seen previously [5]. The previous analyses excluded two bench players who received minimal court time, suggesting the bench players category primarily consisted of in-rotation bench players. The alignment of the current findings for inactive, moderate-vigorous and supramaximal activity with previous research supports the ability for these findings to extend to other professional women’s basketball teams, however, suggests differences in maximal activity might be team-specific.

The differences in match demands found between in-rotation and out-rotation bench players support the notion that when comparing match demands between starters and bench players, bench players should be separated by who is in or out of the main rotation. These findings show that assessing bench players as one group underestimates the demands of in-rotation bench players and overestimates the demands of out-rotation bench players. This idea suggests that comparisons in previous research between starters and bench players might not be optimal, and provides an improved method of player categorization for future research. These findings also have implications for prescribing supplementary conditioning stimuli for bench players to match the activity of starters. Bench players not in the main rotation might need to receive additional conditioning stimuli in all activity categories, while bench players in the main rotation might only need additional moderate-vigorous and maximal activity.

While many differences in match demands were found between player roles, fewer differences were found for training demands (Figure 1 and Figure 2). Average training intensity and volume were not different between player roles. When comparing absolute and relative time spent in each activity intensity category in training sessions, starters performed approximately three more minutes of moderate-vigorous activity, and spent approximately 8% less relative time being inactive than out-rotation bench players. Similar findings have been shown previously where starters experienced greater weekly training demands than bench players in National Collegiate Athletic Association Division 1 men’s basketball [30], suggesting this trend might exist across multiple demographics, divisions, and geographical locations. As the coach’s intention for individualizing training activity between players was not determined, it is possible that this imbalance between starters and out-rotation bench players was intended to better prepare the starting players who were likely to receive more playing time in matches. If the coach intended for all players to perform the same activity in training, the greater duration of moderate-vigorous activity and lower proportion of inactivity for starters could be due to several factors. Firstly, in drill activities, starters might be more experienced and able to perform drills at the prescribed intensity immediately, rather than taking time to watch and learn. Secondly, in gameplay, starters might have greater responsibility for running plays due to their superior ability and experience relative to out-rotation bench players. Lastly, when the number of players at training exceeds the number of players required for gameplay, starters might spend more time on the court while out-rotation bench players spend more time substituted to reflect their relative involvements in competitive matches. These differences in training demands between starters and out-rotation bench players suggest that out-rotation bench players might need to be prescribed extra moderate-vigorous activity in training sessions in addition to topping up their match activity to ensure they receive appropriate conditioning stimuli to meet that of the starting players and maintain their conditioning levels. Additionally, starters might require more recovery practices between training sessions to reduce fatigue and subsequent injury risk.

When comparing match demands between competition levels, no differences were present (Figure 3 and Figure 4). This finding contrasts previous research in men’s basketball where match demands differed between competition levels [11,12]. One study found a higher-level team performed more moderate- and high-intensity activity than a lower-level team, suggested to be due to higher-level teams executing more team-level structures involving all players, with lower-level teams primarily executing plays on an individual level [11]. In another study, the higher-level team performed more jogging and running, but less standing/walking and sprinting, suggested to be due to lower-level players making more errors and subsequently performing more high-intensity transitions between offence and defense [12]. It is possible that both these suggested activity trends occurred in the present study to balance the amount of high-intensity activity performed between competition levels. Alternatively, differences in match demands between competition levels might be typical of men’s basketball, but not women’s basketball. The findings of the present study suggest that in women’s basketball, semi-professional players are adequately prepared to endure the match demands of professional competition, provided the minutes played are similar.

While no differences in match demands were seen between competition levels, some differences were found for sessional training demands (Figure 3 and Figure 4). Average training session intensity was not different between competition levels, while average training session volume was greater for the professional team than the semi-professional team. The professional team also spent more absolute time being inactive and performing light-intensity activity, while the semi-professional team spent more relative time performing moderate-vigorous activity. These findings suggest that while the professional team trainings were of a longer duration, the extra time was spent performing low-intensity activity. Previous research suggests that higher levels of competition might involve more tactical elements and the execution of plays on a team-level [11]. This suggestion might explain the greater volumes of low-intensity activity for the professional team if this reflects time where the coach is explaining tactical plays and providing direction to the players. This rationale is logical due to professional teams generally having more time to train and therefore more time to spend teaching different plays, and having more coaching staff to strategize and implement tactics. Tactical implementation might also take a greater priority in a professional training environment where skill level is higher [31,32] and superior tactics are therefore needed to gain a competitive advantage, contrary to semi-professional competition where time spent developing skills might be more beneficial.

While differences in sessional training demands between competition levels only existed for inactive, light and moderate-vigorous activity, the professional team spent 1.6–2.1× more cumulative weekly time in all activity categories, and experienced twice the average cumulative weekly training volume than the semi-professional team (Figure 5). These findings reflect differences in the weekly training schedule between the teams. Differences in weekly training volumes can have implications for players transitioning from a semi-professional season to a professional season. This transition often occurs in women’s basketball where professional players compete semi-professionally in the off-season to supplement their income, which is typically lower than in men’s basketball [13]. The transition from semi-professional training to professional training could result in large spikes in training demands at the beginning of the professional pre-season, potentially increasing player injury risk [2]. This effect is likely to be amplified if players take a short break between seasons, and by the tendency for pre-season trainings to involve a greater volume of activity than in-season trainings [33]. In order to mitigate the potential increased risk of injury, it might be prudent for coaches to allocate a greater proportion of training time at the beginning of the pre-season to lower-intensity activities such as teaching plays. This approach in combination with appropriate athlete-monitoring techniques might allow semi-professional players to progressively adapt to professional training demands and lower the risk of injury.

While this study investigates several novel aspects of basketball training and match demands, it is acknowledged that these results might not be representative of all teams or competitions. Future research could focus on quantifying training and match demands from multiple teams within the same league, as well as other leagues and geographical locations. Additionally, while the present study provided insight into overall match demands (including stoppages) for training prescription purposes, it could also be of interest to quantify the match demands of live time only. Quantifying the demands of live time would greatly reduce the amount of inactive activity recorded, and could enable training intensity to be prescribed specifically for the demands of live play periods. Furthermore, while no differences were found for average match demands between competition levels, differences in peak demands might exist [34]. Lastly, investigation of on-court movement patterns might provide insight into the causes of differences in training and match activity between player roles and competition levels.

## 5. Conclusions

This study showed that match demands decrease from starters to in-rotation bench players, and from in-rotation to out-rotation bench players, suggesting that both in-rotation and out-rotation bench players might not be prepared for a potential increase in playing time and subsequent increase in match demands. Match demands did not differ between competition levels in women’s basketball, however cumulative weekly training demands were approximately twice as great for the professional team than the semi-professional team. This finding suggests that while semi-professional players might be prepared for professional match demands should they transition to professional competition, they will likely be unprepared for professional training demands. These findings can be used to create more appropriate conditioning programs to improve performance and reduce injury risk, and to ensure players are adequately prepared to move between player roles and competition levels in basketball.

## Figures and Tables

**Table 1:** Participant characteristics.

Team	Players	Age (Years)	Height (cm)	Mass (kg)	Training Sessions Monitored	Matches Monitored
SP Men	13	26.8 ± 5.2	192.3 ± 7.5	96.2 ± 16.4	32	22
SP Women	12	28.1 ± 5.0	176.0 ± 9.7	75.9 ± 18.2	33	21
P Women	12	25.2 ± 5.9	180.6 ± 10.7	79.3 ± 17.1	54	20

SP = semi-professional; P = professional.

**Table 2:** ${\dot{V}O}_{2}$R thresholds and average associated AvF_NET_ thresholds for each intensity band.

Intensity Band	$\dot{V}O_{2}R\;\mathrm{Thresholds}$ (%)	Associated AvF_NET_ Thresholds (N; Mean ± SD)
Inactive	≤10%	≤216 ± 55
Light	>10–40%	>216 ± 55–454 ± 92
Moderate-Vigorous	>40–90%	>454 ± 92–850 ± 174
Maximal	>90–100%	>850 ± 174–929 ± 191
Supramaximal	>100%	>929 ± 191

## Data Availability

The data presented in this study are available on request from the corresponding author. The data are not publicly available due to privacy.
